# The Antiosteoporosis Effects of Zhuanggu Guanjie Pill* In Vitro* and* In Vivo*

**DOI:** 10.1155/2018/9075318

**Published:** 2018-09-23

**Authors:** Li-juan Chai, Yue Zhang, Pan-yang Zhang, Ya-nan Bi, Xiao-mei Yuan, Yu-hong Li, Yan-yan Wang, Lei Song, Li-kang Sun, Kun Zhou

**Affiliations:** ^1^Institute of Traditional Chinese Medicine, Tianjin University of Traditional Chinese Medicine, Tianjin 300193, China; ^2^Tianjin State Key Laboratory of Modern Chinese Medicine, Tianjin 300193, China; ^3^Key Laboratory of Formula of Traditional Chinese Medicine (Tianjin University of Traditional Chinese Medicine), Ministry of Education, Tianjin 300193, China

## Abstract

We investigated the beneficial effects and underlying mechanisms of Zhuanggu Guanjie (ZGGJ) pill in osteoporosis* in vitro* and* in vivo*. Bone marrow macrophages from 4–6-week-old mice were cultured in the presence of macrophage colony-stimulating factor (15 ng/mL) and receptor activator of nuclear factor-*κ*B ligand (30 ng/mL). Osteoclast differentiation was determined by quantification of tartrate-resistant acid phosphatase activity. Gelatin zymography was used to detect the activity of matrix metalloproteinases in osteoclasts. Ovariectomized rats were administered orally with estradiol valerate or ZGGJ for 8 weeks. Blood was collected to measure serum indices. Tibiae were harvested to carry out bone microcomputed tomography scanning, histomorphological analysis, and bone strength determination. ZGGJ inhibited tartrate-resistant acid phosphatase activity, matrix metalloproteinase 9 expression, and bone resorption* in vitro*. At doses of 0.55, 1.1, and 2.2 g/kg, ZGGJ exerted significant osteoprotective effects including inhibition of bone turnover markers and improved tibia bone strength in ovariectomized rats. Microcomputed tomographic analysis showed that ZGGJ improved the trabecular architecture with increased connectivity density and trabecular thickness and decreased trabecular spacing. These results revealed that ZGGJ prevents bone loss induced by ovariectomy in rats and that inhibition of bone resorption is involved in the bone-protective effects of ZGGJ.

## 1. Introduction

Osteoporosis is characterized by a systemic decrease in bone mass and impairment of microarchitecture resulting in fragility fractures, which is a common disease in the aging population, especially postmenopausal women [1]. Hormone replacement therapy is the first-line therapy for prevention and treatment of osteoporosis in postmenopausal women because of estrogen deficiency during menopause, leading to bone loss. However, hormone replacement therapy also has adverse effects after long-term use, such as increased risks of breast cancer and cardiovascular events [2]. Although there are several effective modalities for osteoporosis treatment, continuous efforts are being made to develop novel agents that improve outcomes and minimize the risks of adverse events. In China, traditional Chinese medicines (TCMs) comprising herbal formulas are a growing alternative to prevent osteoporosis [3]. TCMs are attracting the attention of researchers worldwide because of their active and effective properties and lower reported side effects compared with synthetic drugs [4].

Zhuanggu Guanjie (ZGGJ) pill is a TCM formula recorded in the Chinese Pharmacopoeia [5]. The ZGGJ formula consists of 12 herbs including rehmannia radix praeparata (*Rehmannia glutinosa *(Gaetn.)* Libosch. ex Fisch. et Mey.*), fructus psoraleae (*Psoralea corylifolia *Linn.), caulis Spatholobi (*Kadsura interior*), rhizoma drynariae (*Davallia mariesii T.Moore ex Baker*), frankincense (*Boswellia carterii Birdw.*), myrrh (*Commiphora myrrha (T.Nees) Engl.*), parasitic loranthus (*Taxillus sutchuenensis (Lecomte) Danser*), radix dipsaci (*Dipsacales*), rhizoma cibotii (*Cibotium barometz(L.) J.Sm.*), herba epimedii (*Epimedium brevicornu Maxim.*), radix angelicae tuhuo (*Heracleum*), and radices saussureae (*Radix Aucklandiae*). In China, ZGGJ has been used as an osteoarthritis therapy for almost 30 years and is used to treat osteoporosis in some Chinese hospitals with several clinical reports published in Chinese journals. Some studies have suggested that herbs in the ZGGJ formula have beneficial effects in osteoporosis patients, such as rhizoma drynariae [6], radix dipsaci [7], rhizoma cibotii [8], and fructus psoraleae [9–11].

These previous studies imply that ZGGJ has a therapeutic antiosteoporosis action. However, the definitive therapeutic effect and underlying mechanisms of ZGGJ in osteoporosis are unclear. The aim of this study was to investigate the beneficial effects and explore the potential mechanisms of ZGGJ in osteoporosis* in vitro* and* in vivo*.

## 2. Materials and Methods

### 2.1. Drugs and Reagents

Zhuanggu Guanjie pill (Lot: 1410013S) was purchased from Sanjiu Medical & Pharmaceutical Co. Ltd. (Shenzhen, China). Estradiol valerate tablet (Progynova) was purchased from Delpharm Lille S.A.S (Lys-Lez-Lannoy, France). Standard compounds epimedin A, epimedin B, protocatechuic acid, verbascoside, naringin, quercetin, psoralen, isopsoralen, 8-methoxsalen, formononetin, psoralidin, baohuoside I, bavachin, osthole, costundide, bakuchiol, and acetyl-11-keto-*β*-boswellic acid were purchased from Chengdu Pufeide Biological Technology Co. Ltd. Icariin was obtained from the China Food and Drug Testing Institute.

Fetal bovine serum (FBS) was obtained from Bioind. *α*-Minimal essential medium (MEM) and a penicillin-streptomycin solution were obtained from Hyclone (Logan, USA). M-CSF and receptor activator for nuclear factor-*κ*B ligand (RANKL) were purchased from R&D Systems Europe limited (Abingdon, UK). The SensiZyme Cathepsin K Activity Assay Kit was purchased from Sigma (St Louis, USA). Alkaline phosphatase (ALP) kits were purchased from BioSino Bio-Technology and Science Inc (Beijing, China). A tartrate-resistant acid phosphatase (TRAP) kit was purchased from Beyotime Institute of Biotechnology (Jiangsu, China). Osteocalcin, N-terminal propeptide of type 1 procollagen (P1NP), osteoprotegerin (OPG), and RANKL ELISA kits were purchased from CUSBIO Life Science (Wuhan, China). Acetonitrile, methanol, and acetic acid (all HPLC grade) were obtained from Tianjin Fuyv Special Chemicals Co., Ltd.

### 2.2. HPLC

HPLC was performed using a UitiMate 3000 (Thermo Fisher Scientific, USA). Chromatographic separation was carried out on an Agilent Eclipse XDB-C18 (4.6×250 nm, 5 *μ*m) at 35°C with a flow rate of 1 ml/min. The mobile phases consisted of eluent A (acetonitrile) and eluent B (0.1% formic acid in water, v/v). The linear gradient program was as follows: 6%–16% B from 0 to 15 min, 16%–30% B from 15 to 30 min, 30%–36% B from 30 to 35 min, 36%–52% B from 35 to 40 min, 52%–67% B from 40 to 60 min, 67%–75% B from 60 to 65 min, 75%–95% B from 65 to 69 min, 95% B from 69 to 76 min, 95%–6% B from 76 to 77 min, and 6% B from 77 to 80 min. The injection volume was 10 *μ*L, and the detection wavelength was 254 nm.

### 2.3. Animals

Sprague-Dawley rats were purchased from Beijing HFK Bioscience Technology Co. LTD (Beijing, China). The rats were housed in the Laboratory Animal Centre of Tianjin University of Traditional Chinese Medicine. Rats were provided with a standard diet and water ad libitum. They were acclimated to the experimental conditions of 18–23°C and 50%–65% humidity. The protocols and operations were performed in accordance with regulations on the administration of laboratory animals issued by the Ministry of Science and Technology of China. Experiments were approved by the Laboratory Animal Ethics Committee of Tianjin University of Traditional Chinese Medicine (Permit Number: TCM-LAEC 2014011).

### 2.4. Measurement of ALP Activity in Osteoblasts

MC3T3-E1 cells were seeded in 96-well plates and cultured for 24 h in *α*-MEM with 10% FBS, penicillin (100 U/mL), and streptomycin (100 *μ*g/mL). Then, the cells were treated with ZGGJ for 72 h. The medium was removed, and the cells were washed twice with PBS. Lysis buffer (50 *μ*L) was added to each well, followed by incubation at 37°C for 15 min, and then carbonate buffer was added, followed by incubation at 37°C for 5 min. A benzene disodium phosphate solution (50 *μ*L; prewarmed at 37°C) was added to each well, mixed, and then incubated at 37°C for 45 min in a water bath. After K_3_[Fe(CN)_6_] was added and mixed, optical absorbance was measured at 510 nm on a microplate reader.

### 2.5. Osteoclast Differentiation from Bone Marrow Cells

Bone marrow cells were prepared by obtaining bone marrow from the femora and tibiae of 4–6–week-old KM mice. In brief, bone marrow was flushed out with *α*-MEM supplemented with 15% FBS and 1% penicillin-streptomycin solution from the femora and tibiae of mice using a 26 G needle. Bone marrow cells were washed with *α*-MEM and then cultured for 24 h to allow attachment of stromal cells at 37°C with 5% CO_2_ in 75-cm^2^ flasks containing *α*-MEM. Nonadherent cells were collected and resuspended at 5×10^6^ cells/mL in *α*-MEM and incubated for 48 h to allow attachment of osteoclast precursors. Then, the medium was removed, and the cells were washed twice with D-Hank's balanced salt solution. The attached cells were considered as osteoclast precursors.

### 2.6. Measurement of TRAP Activity in Osteoclasts

Osteoclasts were collected on day 8 to measure enzymatic activity. TRAP activity was measured after rinsing cells twice with PBS. In brief, 30 *μ*L of 0.1% Triton X-100 was used to lyse the cells for 10 min. Then, 100 *μ*L of substrate solution (0.5 g p-nitro-disodium phenylphosphate and 1.9 g sodium L-tartrate in 250 mL deionized water; pH 5.2 adjusted with 5 mol/L NaOH) was added to each well in a 96-well plate and incubated at 37°C for 30 min. To stop the reaction, 100 *μ*L of 1 mol/L NaOH was added to each well. The samples and standards were diluted in 20 mmol/L NaOH. Optical absorbance was measured at 405 nm on a microplate reader (Victor™ X5; Perkin Elmer). The nanomolar concentration of p-nitrophenol in each well was calculated.

### 2.7. Gelatin Zymography of MMPs and Pit Formation Assay

Culture supernatants were collected and used to detect MMP9 activity by gelatin zymography [12]. The sample was separated by electrophoresis on a 7.5% SDS-polyacrylamide gel copolymerized with 1% gelatin. After electrophoresis, the gel was washed twice in 2.5% Triton X-100 and incubated in 1% Triton X-100 and 5 mM CaCl_2_ at 37°C for 36 h. The gels were stained with 0.1% Coomassie blue R-250 and destained in 10% acetic acid/H_2_O. MMPs were detected as transparent bands on the blue background of the Coomassie blue-stained gel. Signals were detected using a GENE Genius Bio Imaging System (SynGene).

Osteoclast precursors were seeded in a 96-well microplate at 5×10^4^ cells per well in which one bone slice had been placed. After 48 h of incubation, the medium was replaced with fresh medium with or without ZGGJ. The culture medium was replaced every 3 days. The cells were cultured for 15 days. The bone slices were then fixed with 2.5% glutaraldehyde for 7 min and ultrasonicated in a 0.25 mol/L NH_4_OH solution to remove attached cells. Then, the bone slices were dehydrated through a graded alcohol series (100%–30%) and stained with 1% toluidine blue to visualize resorption pits. Images of each bone slice were captured under a stereo microscope with apochromatic Optics (LEICA S8 APO; LEICA). The number of resorption pits and resorption area were calculated.

### 2.8. Ovariectomy and Treatments

The classical and widely used ovariectomized rat model was adopted. Eight-week-old rats were ovariectomized to establish the experimental osteoporosis model. Twelve rats with a sham operation were used as the control. After 4 weeks, the surviving ovariectomized rats were randomly divided into six groups: model group (treated with water), estradiol valerate group (0.15 mg/kg), and three ZGGJ groups (0.55, 1.1, and 2.2 g/kg crude powder dosages) with 12 rats in each group. The pills were crushed into powder and dissolved in water. Rats were intragastrically administrated once every day for 8 weeks. Control and model group rats were treated with water. The rats were anesthetized and then sacrificed after 12 h of fasting to avoid the effects of food intake on biochemical indices. Blood was collected to calculate serum indices, the tibia for bone histomorphology analysis and microcomputed tomography (micro-CT), and the femur to measure bone strength.

### 2.9. Analysis of Serum Biochemical Indices

Serum ALP, Ca, and P were measured using a 7020 biochemical analyzer (Hitachi, Japan) and kits (BioSino, Beijing, China). Serum TRAP was measured using a TRAP kit (Beyotime Institute of Biotechnology), according to the manufacturer's protocol. Serum osteocalcin, P1NP, OPG, and RANKL were measured using ELISA kits (CUSBIO Life Science).

### 2.10. Bone Micro-CT Analysis

The right tibia was soaked in 75% ethanol for 72 h. The ethanol was replaced once every 24 h. Then, the tibia was fixed in anhydrous ethanol for bone densitometry using a vivaCT 40 Micro-CT scanner (SCANCO Medical AG, Zurich, Switzerland). A CT scan from the proximal tibia, where the epiphyseal growth plate had disappeared, to its distal end was performed. The number of slices was 80 of 10.6 *μ*m in thickness, so that the region of interest (ROI) was 0.84 mm from the epiphysial line to the distal tibia. Images were acquired at 70 kVp and 114 mA with a 200 ms integration time. A three-dimensional (3D) model was reconstructed, and structural evaluations were performed using SCANCO Medical software. An irregular anatomical ROI adjacent to the periphery of the cortical boundary drawn using a manual algorithm was adopted. The degree of anisotropy (DA) was the ratio of the longest and shortest vectors of the mean intercept length. Bone volume (BV) was the material volume of bone, and total volume (TV) was the apparent total volume of bone including the volume of the marrow cavity in the bone. Bone density is shown as the densities of TV and BV. BV/TV, trabecular connectivity density (Conn.D), trabecular number (Tb.N), trabecular thickness (Tb.Th), and trabecular separation (Tb.Sp) were calculated by measuring 3D distances without a model.

### 2.11. Bone Histomorphological Analysis

The left tibia was fixed with 10% buffered formalin, decalcified with 8% formic acid and 8% oxalic acid, and embedded in paraffin for sectioning at 5 *μ*m. Sections were stained with hematoxylin and eosin (H&E). Histomorphological examination was performed using an optical microscope (BX51, Olympus). Images were captured by an electronic camera system (DP71, Olympus).

### 2.12. Bone Strength Analysis

Bone strength of the tibia was measured using a YLS-16A small animal bone strength analyzer (Jinan Yiyan Technology Co. Ltd., Jinan, China). The maximum load and maximum force (in grams) applied to the tibia until fracture was tested in mode 1. The tibia was placed horizontally on the supporting frame and fixed. The three-point bend method was applied, two of which were ~8 mm and the actuator acted in the middle of the space at 1.3 mm/s. The result was the maximum lateral load of the tibia. In addition, the structural strength was tested in mode 2 in which the tibia of the other side was placed on a pedestal that was smooth to ensure the bone could be crushed, and the actuator was also applied at 1.3 mm/s. The data of bone strength were transformed to be shown as a unit of force.

### 2.13. Statistical Analysis

Data are shown as the mean ± SEM. Statistical analysis was performed by one-way ANOVA. P < 0.05 was considered as significant.

## 3. Results

### 3.1. HPLC Fingerprint of ZGGJ

**Figure 1:**
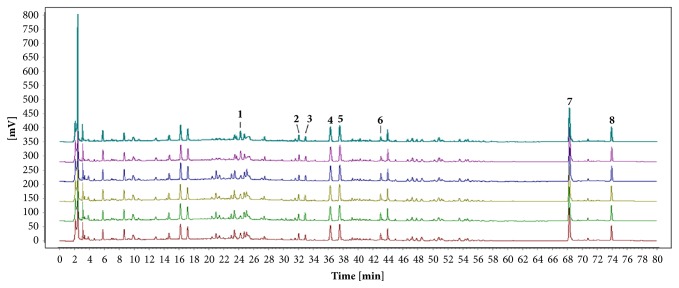
HPLC fingerprints of six ZGGJ samples. Peak 1, naringin; peak 2, quercetin; peak 3, icariin; peak 4, psoralen; peak 5, isopsoralen; peak 6, baohuoside I; peak 7, bakuchiol; peak 8, acetyl-11-keto-*β*-boswellic acid.

HPLC analysis was demonstrated to be precise and sensitive by analyzing the relative standard deviation (RSD) of the relative retention time (RRT) and relative peak area (RPA). The RSD of both RRT and RPA was ≤1.99%, indicating that the method was precise and sensitive enough to evaluate ZGGJ. The HPLC fingerprints of ZGGJ samples were obtained and compared with a reference sample using the software package Similarity Evaluation System for Chromatographic Fingerprint of Traditional Chinese Medicine (Version 2012) from the China National Pharmacopoeia Commission. The samples all had similar HPLC profiles with similarity values from 0.984 to 0.999, and eight of the peaks were identified as naringin, quercetin, icariin, psoralen, isopsoralen, baohuoside I, bakuchiol, and acetyl-11-keto-*β*-boswellic acid by comparing their retention time and UV spectrum with those of reference samples (Figure 1).

### 3.2. ZGGJ Has No Significant Effect on ALP Activity* In Vitro*

**Figure 2:**
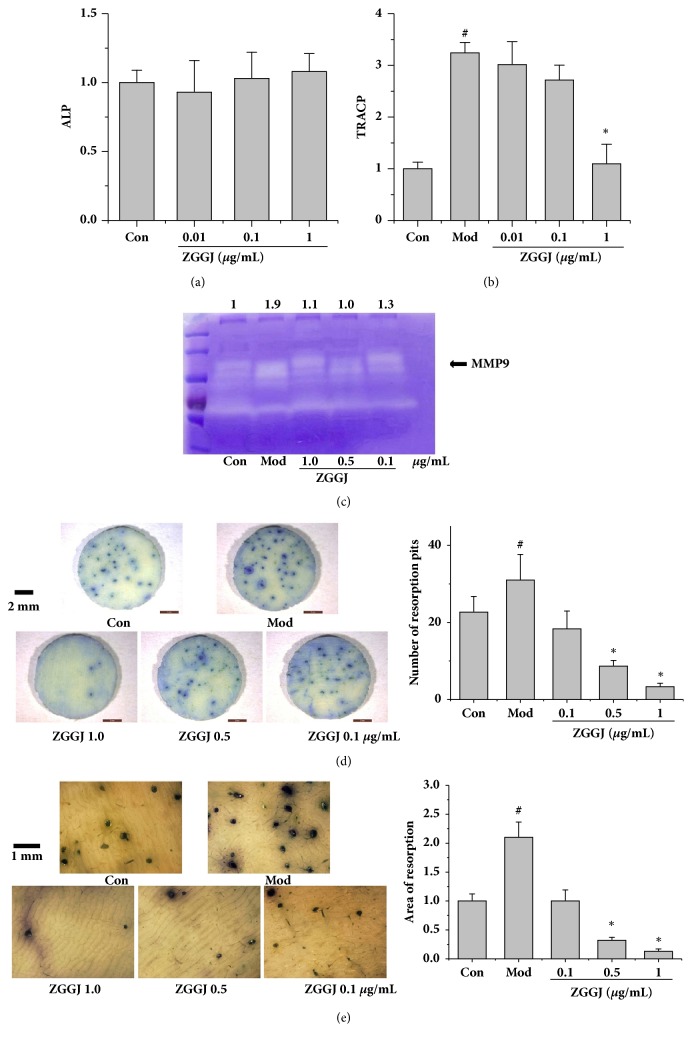
ZGGJ inhibits bone resorption and MMP9* in vitro*. (a) ZGGJ had no significant effect on ALP activity* in vitro*. (b) ZGGJ reduced osteoclast TRACP activity* in vitro*. (c) MMP9 detected by gelatin zymography. (d) Representative photomicrographs (×9) of bone slices and numbers of resorption pits. (e) Representative photomicrograph (×40) of bone slices and total areas of resorption pits. Con, control group with cells cultured in medium without M-CSF or RANKL; Mod, model group with cells cultured in the presence of 15 ng/mL M-CSF and 30 ng/mL RANKL; ZGGJ, groups treated with various doses of ZGGJ. ^#^*p* < 0.05 versus Con; *∗p* < 0.05 versus Mod.

The effect of ZGGJ on ALP activity in osteoblasts was investigated* in vitro*. Compared with the control group, cells treated with ZGGJ exhibited slightly elevated ALP activity, but without a significant difference, which implied that ZGGJ had negligible effects on osteoblasts (Figure 2(a)). Therefore, we focused on osteoclasts.

### 3.3. ZGGJ Reduces TRAP Activity in Osteoclasts* In Vitro*

To evaluate the influence of ZGGJ on TRAP activity in osteoclasts, bone marrow macrophages from 4–6-week-old KM mice were cultured in the presence of 15 ng/mL M-CSF and 30 ng/mL RANKL. After 8 days of treatment, osteoclast differentiation was determined by quantification of TRAP activity. ZGGJ induced a significant decrease in TRAP activity at a dose of 1 *μ*g/mL (Figure 2(b)).

### 3.4. ZGGJ Inhibits MMP9 and Bone Resorption* In Vitro*

Gelatin zymography was used to detect the activity of MMP9 in osteoclasts. ZGGJ significantly attenuated MMP9 expression that was significantly increased in the model group by M-CSF and RANKL (Figure 2(c)).

To evaluate the direct effects of ZGGJ on bone resorption by osteoclasts, bone marrow macrophages induced by M-CSF and RANKL were seeded on bovine bone slices. After treating osteoclasts seeded on the bone slice for 16 days, we detected the effect of ZGGJ on bone resorption activity. After 16 days of culture, the amount of bone lacunas was increased significantly in the model group, and 0.5 and 1.0 *μ*g/mL ZGGJ groups showed significant decreases in the formation of bone lacunas compared with the model group. The area of bone lacunas in bone slices was also analyzed to evaluate the effect of ZGGJ on bone resorption by osteoclasts. After 16 days, bone lacuna areas in bone slices of the model group were increased significantly, and a significant decrease in bone lacuna areas was observed in 0.5 and 1.0 *μ*g/mL ZGGJ groups (Figures 2(d) and 2(e)).

### 3.5. ZGGJ Ameliorates Body Weight and Serum Biochemical Indices in Ovariectomized Rats

**Figure 3:**
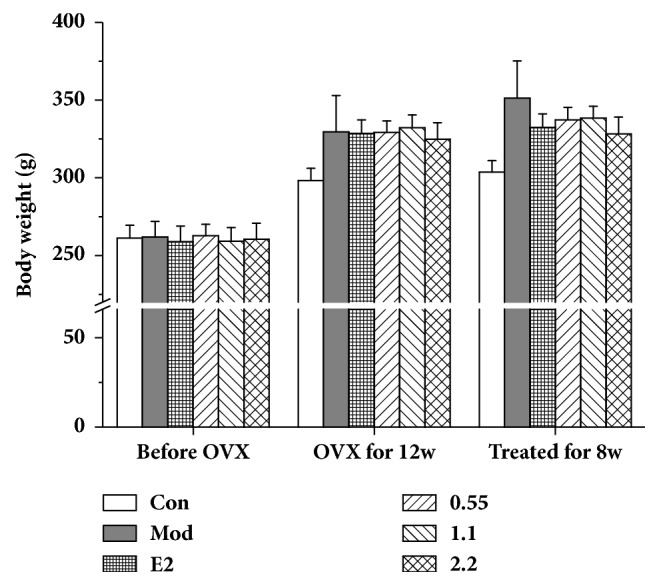
Effects of ZGGJ on body weight. Con, control group; Mod, model group; E2, 0.15 mg/kg estradiol valerate group; ZGGJ, groups treated with various doses of ZGGJ.

**Figure 4:**
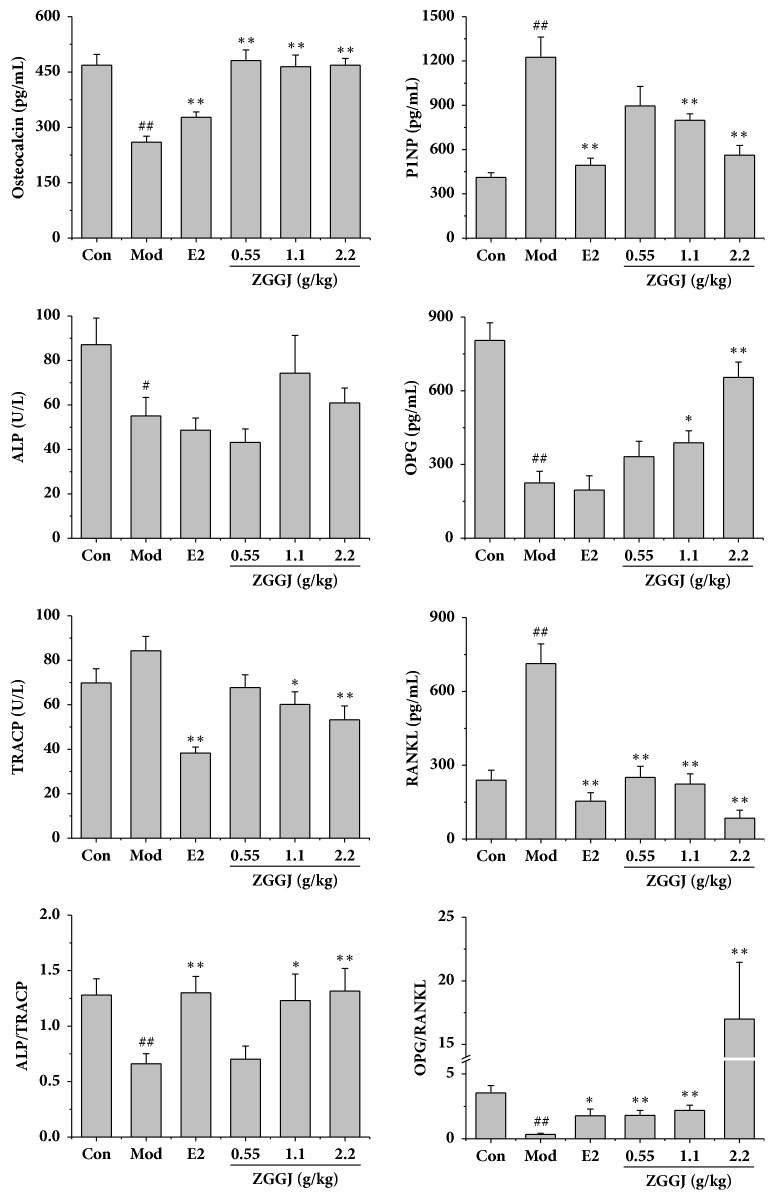
Effects of ZGGJ on serum indices. Con, control group; Mod, model group; E2, 0.15 mg/kg estradiol valerate group; ZGGJ, groups treated with various doses of ZGGJ. ^##^*p* < 0.01 versus Con; *∗p* < 0.05 and *∗∗p* < 0.01 versus Mod.

Before the ovariectomy, the body weights of rats in all groups were similar. At 12 weeks after ovariectomy, the body weight of ovariectomized rats was higher than that of control rats. After treatments for 8 weeks, the body weights of rats treated with ZGGJ or estradiol valerate were lower than those of model rats, but still higher than those of the control. However, no significant differences were observed (Figure 3). Some serum markers were analyzed, including osteocalcin, P1NP, ALP, OPG, TRACP, and RANKL. The serum osteocalcin level of the model group was decreased greatly compared with that of the control group, which was increased markedly in rats of the three ZGGJ groups and the 0.15 mg/kg estradiol valerate group compared with that of the model group. After 8 weeks of ZGGJ treatment, serum osteocalcin levels had recovered to normal. Serum ALP and OPG of the model group were both significantly lower than those of the control group. Compared with model group, OPG was strongly increased after 1.1 and 2.2 g/kg ZGGJ administrations, while ALP levels of the three treatment groups showed no significant differences. Serum TRAP and RANKL of the model group were significantly higher than those of the control group, and their activities in rats of the ZGGJ groups were lower than those in the model group, which tended to be normal (Figure 4). These results suggest that ZGGJ inhibits bone resorption in rats. Interestingly, the P1NP level of the model group was higher than that of the control group, which is secreted by osteoblasts during bone formation.

### 3.6. ZGGJ Ameliorates the Bone Microstructure in Ovariectomized Rats

**Figure 5:**
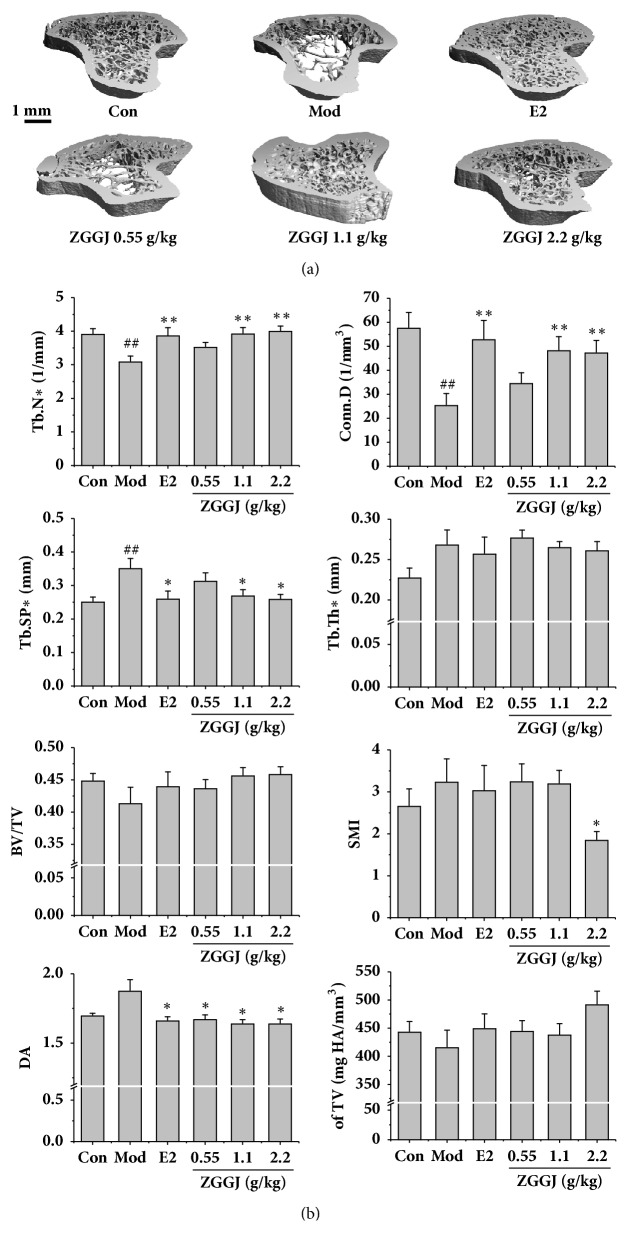
Micro-CT images and effects of ZGGJ on Conn.D, Tb.N, Tb.Th, and Tb.Sp. (a) Micro-CT images. (b) Changes of Conn.D, Tb.N, Tb.Th, and Tb.Sp. Con, control group; Mod, model group; E2, 0.15 mg/kg estradiol valerate group; ZGGJ, groups treated with various doses of ZGGJ. ^##^*p* < 0.01 versus Con; *∗p* < 0.05 and *∗∗p* < 0.01 versus Mod.

There were no differences in tibial lengths between treatment groups in micro-CT scanning images. The trabecular bone was rare at the area located 1.2 mm below the growth plate in the proximal tibia of normal rats. Thus, the area less than 1 mm from the growth plate to the distal direction was selected as the ROI. The 3D morphometric evaluation of the trabecular ROI revealed a good therapeutic effect on the trabecular bone mineral density after treatment, and the therapeutic effect was dose dependent (Figure 5(a)). Compared with the control group, Tb.N and Conn.D of the model group were decreased significantly, which both recovered to near normal levels after treatment for 8 weeks, although the effect in the 0.55 g/kg ZGGJ group was less than those in 1.1 and 2.2 g/kg ZGGJ groups. Tb.Sp and DA of the model group returned to normal after treatment, which were higher than those of the control group. Tb.Th, BV/TV, SMI, and TV were all changed, but had no significant differences (Figure 5(b)).

**Figure 6:**
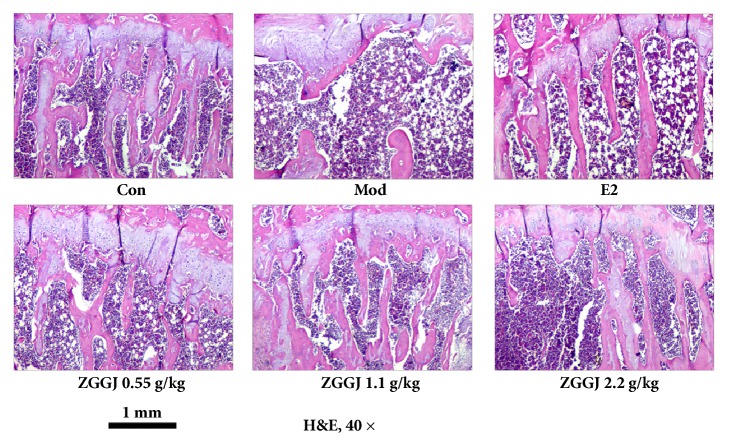
Representative images of histomorphology. Con, control group; Mod, model group; E2, 0.15 mg/kg estradiol valerate group.

Histomorphology showed that the number of trabecular bones was significantly reduced in the proximal tibia of model group rats compared with the control group, and the trabecular bone had almost disappeared in the region around the epiphyseal growth plate. After treatment, bone formation under the periosteum and the number of trabecular bones were increased, and the gap between trabecular bones was decreased by various degrees (Figure 6).

### 3.7. ZGGJ Enhances Bone Strength of the Tibia in Ovariectomized Rats

**Figure 7:**
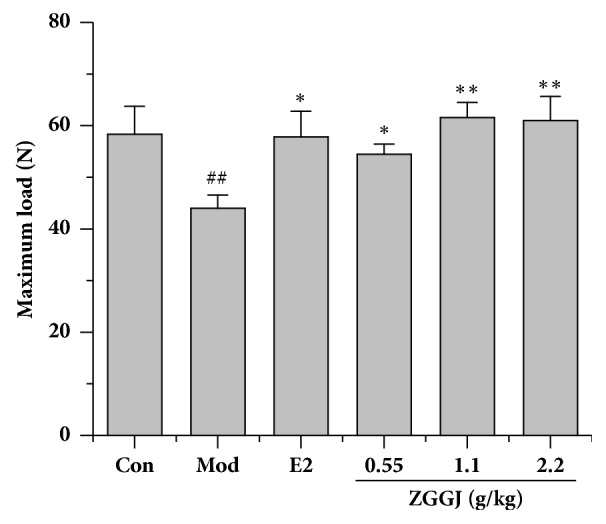
ZGGJ enhances bone strength of the tibia. Con, control group; Mod, model group; E2, 0.15 mg/kg estradiol valerate group; ZGGJ, groups treated with various doses of ZGGJ. ^##^*p* < 0.01 versus Con; *∗∗p* < 0.01 versus Mod.

Bone strength is an important parameter related to fracture risk. The right tibia was used to determine bone strength. Unsurprisingly, bone strength of model group rats was significantly lower than that of the control, and bone strength was significantly increased after treatment (Figure 7), indicating that ZGGJ protects against osteoporotic fracture.

## 4. Discussion

This study evaluated the effects of ZGGJ on osteoporosis* in vitro* and* in vivo*. ZGGJ inhibited bone resorption, which correlated with TRAP and MMP9 expression* in vitro*, demonstrating direct inhibitory effects on osteoclast resorption. Our results showed that ZGGJ at doses of 0.55, 1.1, and 2.2 g exerted significant osteoprotective effects. Micro-CT analysis showed that ZGGJ improved the trabecular architecture as indicated by increases in Conn.D and Tb.N, and a decrease in Tb.Sp. Furthermore, administration of ZGGJ improved bone strength of the tibia. These results indicate that ZGGJ has direct beneficial effects on ameliorating osteoporosis in ovariectomized rats, and the mechanism may be mediated by inhibiting bone resorption.

TRAP is secreted by osteoclasts, which reflects the osteoclast number and activity, and serum TRAP activity correlates with resorptive activity in bone metabolism disorders [13]. RANKL, an osteoclastogenic cytokine of the tumor necrosis factor family secreted by osteoblastic cells [14], promotes osteoclast formation and plays an important role in bone metabolism. Serum ALP, osteocalcin, and P1NP are serum markers that reflect osteoblast activities including bone formation [15].

To evaluate the influence of ZGGJ on osteoblasts, we investigated their activities and indices. ALP activity of osteoblasts treated with ZGGJ showed no significant differences from the control group* in vitro*. The serum levels of ALP and osteocalcin were markedly decreased in the model group compared with the control group* in vivo*. Osteocalcin activity was increased greatly after oral administration of ZGGJ. However, ALP levels of rats in the three groups treated with ZGGJ showed no remarkable differences from the model group, which may be due to ZGGJ decreasing bone resorption rather than increasing bone formation. However, P1NP, which is another important marker of bone formation [16], showed an increasing trend in ovariectomized mice. Ryu et al. [17] reported similar results in castrated rats in which P1NP increased gradually after orchiectomy, the generally accepted approach to induce osteoporosis in male rats. Moreover, a clinical study suggested a higher rate of hip fracture in both men and women when the P1NP level increases to >60 *μ*g/L. We cannot yet explain the phenomenon, but it is encouraging that administration of ZGGJ normalized the P1NP level. The above results imply that ZGGJ has a negligible effect on osteoblasts.

A previous study indicated that bone loss caused by estrogen deficiency is mainly attributed to an increase of osteoclastic bone resorption [18]. Our results suggest that ZGGJ has a positive effect on bone metabolism by inhibiting bone resorption and potential antiosteoporotic effects on osteoblasts and ovariectomized rats. Compared with the model group, the TRAP and MMP9 activities of osteoblasts pretreated with ZGGJ were decreased significantly, because ZGGJ inhibited osteoclastogenesis* in vitro*. Furthermore, in vivo results indicated that RANKL and TRACP activities were significantly enhanced in the model group in comparison with the control group. Moreover, oral administration of ZGGJ for 8 weeks lowered serum TRAP activity and RANKL levels, suggesting that ZGGJ prevents the ovariectomy-induced increase of bone resorption in rats.

Micro-CT is the most effective and sensitive tool to detect early bone changes compared with dual X-ray absorptiometry, peripheral quantitative computed tomography, and magnetic resonance imaging [19]. We measured various parameters by micro-CT, including Conn.D, Tb.N, Tb.Th, and Tb.Sp, which have been successfully used to assess the microarchitecture of rodent trabecular bones [20]. Osteoporosis is accompanied by a low bone mass density and changes in the microstructure of bone tissue, such as reductions in Tb.Th, Tb.N, and Conn.D, and an increase in Tb.Sp [21, 22]. These characteristics of osteoporosis were observed in the model group, whereas ZGGJ dose-dependently increased Conn.D and Tb.N and decreased Tb.Sp. The effects of high ZGGJ doses were similar to those of estradiol valerate, a commonly prescribed drug for osteoporosis treatment.

A serious consequence of osteoporosis is fracture [23]. Therefore, the fracture resistance of bone is important to evaluate treatment effects for osteoporosis. To accurately predict the fracture risk, we combined micro-CT analysis, histomorphology, and bone strength analysis to evaluate bone quality that is important to assess drug treatments for osteoporosis.

To provide an intuitive approach to predict fracture risk in the current study, we analyzed bone strength of the femur. The femur is the major site for fractures in osteoporosis patients [24, 25]. Based on the results of bone biomechanical strength testing, ZGGJ treatment significantly improved bone strength in ovariectomized rats compared with the model group.

Bone resorption is mediated by osteoclasts, which results in bone loss by eliminating the mineralized matrix in bone. It has been well reported that excessive bone resorption is related to bone loss diseases such as osteoporosis and rheumatoid arthritis. Therefore, we examined the inhibitory effect of ZGGJ, a well-known TCM for osteoporosis, on bone resorption by osteoclasts and its underlying enzymatic activity. Osteoclast precursor cells originate from hematopoietic stem cells [26] and differentiate into mature multinucleated osteoclasts under the regulation of RANKL and macrophage colony-stimulating factor (M-CSF) [27].

We hypothesized that suppression of RANKL-induced osteoclastogenesis and inhibition of osteoclasts by ZGGJ may be associated with inhibition of TRAP, MMP9, and cathepsin K activities. To test this hypothesis, we first investigated the effect of ZGGJ on RANKL-induced TRAP activity in osteoclasts. In our primary osteoclast culture, we found an increase in TRAP activity induced by M-CSF and RANKL. ZGGJ had direct effects on osteoclast marker enzyme TRAP and the activity of MMP9. ZGGJ also significantly decreased the number and areas of bone lacunas compared with the model group. The inhibition of bone resorption was involved in the bone-protective effects of ZGGJ. However, the exact mechanism, effective components, compatibility law, and optimization of ZGGJ still require further research.

## 5. Conclusion

Our study demonstrates that ZGGJ exerts a protective effect against bone loss in ovariectomized rats. We inferred that the drug may have a more potent effect on inhibiting bone resorption rather than promoting bone formation. ZGGJ may be a potential therapeutic agent for osteoporosis.

## Data Availability

The data used to support the findings of this study have not been made available because of its commercial confidentiality. The drug we used in this work belonged to a pharmaceutical company which is patented. However, the preliminary data can be sent upon request to the author of correspondence with permission of its manufacturer.
